# Government Epidemic Prevention and Economic Growth Path Under Public Health Emergency: Theoretical Model and Simulation Analysis

**DOI:** 10.3389/fpubh.2021.748041

**Published:** 2021-09-13

**Authors:** Zhichao Yin, Xiaoxu Chen, Zongshu Wang, Lijin Xiang

**Affiliations:** ^1^School of Finance, Shandong University of Finance and Economics, Jinan, China; ^2^Chow Yei Ching School of Graduate Studies, City University of Hong Kong, Hong Kong, SAR China

**Keywords:** public health emergency, epidemic prevention, economic growth path, theoretical model, simulation analysis

## Abstract

This paper constructs a partial equilibrium model under public health emergency shocks based on economic growth theory, and investigates the relationship between government intervention and virus transmission and economic growth path. We found that both close contacts tracing measures and isolation measures are beneficial to human capital stock and economic output per capita, and the effect of close contact tracing measures is better than that of isolation measures. For infectious diseases of different intensities, economic growth pathways differed across interventions. For low contagious public health emergencies, the focus should be on the coordination of isolation and tracing measures. For highly contagious public health emergencies, strict isolation, and tracing measures have limited effect in repairing the negative economic impact of the outbreak. The theoretical model provides a basic paradigm for the future researches to study economic growth under health emergencies, with good scalability and robustness.

## Introduction

At present, the global economy is still in the shadow of COVID-19. In this unprecedented global public health emergency, the economies of many countries are in recession. The epidemic has brought great trauma to both the production and demand sides of the world economy, and it is difficult for the world to recover to the pre-epidemic level in the short term.

Since the outbreak of COVID-19, the global response to the epidemic and the differences in economic performance provide a window for us to observe the effectiveness of epidemic prevention measures and economic growth. Based on this, we construct a theoretical model to explore the effects of isolation of infected persons and tracing of close contacts on the spread of the epidemic and its impact on economic growth, in order to find the path of economic growth and the government's epidemic prevention plan under the emergency.

This paper finds that epidemic prevention measures have positive effects on economic recovery. On the one hand, both the close contact tracing measure and isolation measures are beneficial in reducing the spread of the virus and contribute to the human capital stock and economic output per capita, but the effect of the close contact tracing measures are better than that of the isolation measures. On the other hand, the paths of economic growth under epidemic prevention measures differ for different intensities of infectious diseases. Under low contagious emergencies, more attention should be paid to the coordination of isolation and tracking measures. For viruses with high contagious, the effects of isolation and tracking measures show attenuation in extreme cases.

There are two marginal contributions in this paper. One is that we construct a model of economic growth in an infectious disease setting by combining an epidemiological model with a Solow growth model, which provides the basic idea for the future researches to study the path of economic growth under an unexpected epidemic. Second, we simulate the epidemic prevention effects and economic effects of isolation measures and tracing measures which commonly used by governments under an epidemic, weighing the optimal policy combinations on the two issues of epidemic prevention and economic growth.

## Literature Review

We review related studies from following aspects: (i) economic impact of public health emergencies; (ii) mechanism of public health emergencies affecting economic growth; and (iii) epidemic prevention measures of public health emergencies.

### Economic Impact of Public Health Emergencies

With the frequent outbreaks of Hog cholera virus, Avian Influenza virus and Ebola virus in recent years, the economic impact and governance of unexpected outbreaks have become hot topics. While viruses pose a threat to human health, they also pose a serious threat to economic and social development. Typical studies such as Gallup and Sachs (1) analyzed the relationship between poverty and malaria for tropical and subtropical countries and found that malaria is an important cause of chronic poverty in these regions. Malaria undermines the domestic economic base and reduces foreign investment, that many malaria-prone countries have an average annual GDP per capita growth rate of <1.3 per cent, and that a 10 per cent reduction in malaria would increase economic growth by 0.3 per cent. Through comparative studies, they found that tropical and subtropical countries with high incidence of malaria would generally experience faster economic growth than their neighbors in the 5 years following malaria elimination. In the face of long-standing epidemics, efficient measures are key to controlling the epidemic. Garner et al. (2) combined epidemiological and economic approaches to assess the economic impact of three infectious diseases, Hog cholera virus, Nipah virus, and Porcinere productive and respiratory syndrome virus, on the Australian pig industry. Local outbreaks of infectious diseases were found to reduce the economic income of the local pig farming industry by 16–37%, and if any of the three diseases spread to a national level, it would reduce the annual income of the national pig farming industry by 5–11%, and cause social and economic disruption. Holtkamp (3) also assessed the impact of the Hog cholera virus outbreak on the U.S. economy from 2005 to 2010 using agribusiness data disclosed by the U.S. Department of Agriculture, and showed that the average annual economic loss due to the outbreak was approximately 664 million dollars. In light of this, they called on quarantine authorities to manage the risk by implementing appropriate measures, including restricting the importation of specific products and isolating and eliminating the source of infection. Joo et al. (4) studied the losses in the Korean tourism industry after the MERS outbreaks from 2015 to 2016. The study found that the outbreak reduced the size of tourists by ~2.1 million and the direct losses to the Korean tourism industry were ~2.6 billion dollars, with associated losses to the accommodation, food and beverage, and transportation sectors of 542 million dollars, 359 million dollars, and 106 million dollars, respectively, which were devastating to the overall Korean economy.

For the economic impact of Covid-19, Burger and Calitz (5) find that Covid-19 exacerbated South Africa fiscal position. While higher government spending helped to restore GDP and maintain debt/GDP stability, the empirical study found that the public spending/GDP ratio exceeded the level at which an increase in that ratio would have positively affected growth. For the Covid-19 epidemic, isolation is one of the important measures. Yezli and Khan (6) find that Iran was not sufficiently protected during the epidemic and many laborers continued to work in order to survive, which increased the spread of the epidemic in Iran. Mugaloglu et al. (7) find that Covid-19 increased the uncertainty of the Turkish economy, with industrial production declined by 8.2%, with losses of 18 and 25% for capital and intermediate goods, respectively.

### Mechanism of Public Health Emergencies Affecting Economic Growth

The occurrence of epidemic diminishes the ability to work and has an impact on economic growth by affecting human health and income.

For the perspective of labor income, infectious diseases directly exacerbate poverty and increase the burden on society. Chronic illness, impaired health and even disability lead to reduced productivity, creating a vicious cycle of poverty and disease, which has been called the “Infectious Diseases of Poverty” (IDoP) by academics. Most of the world's economies have grown by leaps and bounds over the centuries, but more than one-sixth of the world's population remains chronically poor, and this widespread and persistent poverty is tied to infectious diseases (1, 8). Numerous authoritative studies have shown that extreme poverty is often associated with endemic infectious diseases. Poverty induces the occurrence and spread of diseases, and the poverty trap driven by diseases further restricts economic development (9–12). The cost of treating diseases forces families to pay large amounts of money for medicine, and most poor families in less developed countries rarely have major medical insurance and often have to sell what they have, such as farming equipment and livestock, or even mortgage their lands, to pay for medical care and keep up with survival expenses, resulting in the depletion of household assets or even the creation of debt. This rapid loss of productive assets due to epidemics exacerbates the persistence of poverty, as poverty remains difficult to escape from after the epidemic ends due to lack of labor tools and labor capital (e.g., seeds, fertilizers, etc.), and the environmental and health problems caused by poverty in turn provide the conditions for new outbreaks (13). Bonds et al. (14), Ngonghala et al. (15) combine economy and disease on the basis of an infectious disease model and find that in a poverty trap, initial economic and epidemiological conditions determine the long-term trajectory of a society's health and economic development, and that government commitment to improving population health status is one of the necessary conditions for moving out of poverty.

From the perspective of production, infectious diseases weaken human capital accumulation and constrain the level of productivity. Economic activity requires human resources, especially human capital, and the impact of viruses on the health status of human capital will eventually be transmitted to the economic production process (16, 17). Premature death and disability caused by infectious diseases shorten human life and lead to lower levels of human capital, and long-term infectious diseases also reduce the life expectancy of the population, thus further reducing society's investment in human capital, a phenomenon that is prevalent in some low-income countries where AIDS and malaria are endemic (18–20). Fortson (21) examined the impact of the AIDS epidemic on human capital investment and economic growth in Africa. The decline in educational attainment is found to be relatively greater in areas with higher rates of HIV infection, and HIV reduces human capital investment through its effect on longevity. He found that for every 10% increase in HIV transmission, children's schooling would be shortened by half a year, and combined with perennially low school enrollment and graduation rates, slow human capital accumulation drags down African economies. Manuelli (22) examines the long-term impact of combating AIDS and malaria in Africa by constructing a model of human capital accumulation that includes exposure to specific diseases and educational inputs. He finds that if current HIV transmission and malaria rates were reduced by another half, individual worker output would increase by 20–50%, and that improvements in the infectious disease environment would have a positive impact on higher human capital investment. A theoretical analysis by Goenka and Liu (23) using an endogenous growth model that includes human capital found that there are multiple equilibrium growth paths for economic growth, human capital accumulation, and infectious disease incidence. The constraint of infectious diseases on human capital accumulation is an important reason why many less developed countries currently struggle to achieve economic growth or even fall into the poverty trap.

### Epidemic Prevention Measures of Public Health Emergencies

Tthe measures that governments can take generally fall into two categories. One is isolation for controlling the source of infection, which is quick and effective for outbreaks such as foot-and-mouth disease, swine fever, hog cholera, and SARS. While the other is subsidies or transfers to infected populations, which has been more effective in improving poverty levels in areas where AIDS and malaria have been endemic for a long time and in ensuring human capital accumulation.

For government policies during the epidemic, most countries adopted measures such as restricting population movement and maintaining social distance, which helped to control the epidemic but also led to stagnant business production, declining household income, food shortages in some countries (24), and even triggered inflation, financial risks, and local economic crises (25, 26). In China, Covid-19 is a major epidemic in the country in the last decade. China has included Covid-19 into the national category B infectious diseases and managed them according to category A. The nationwide epidemic prevention and control system has responded rapidly (27). Yang et al. (28) integrated population migration data and epidemiological data into a classical epidemiological prediction model to predict and simulate the development of the epidemic, and found that if the control measures were delayed by 5 days, the epidemic scale in China would increase up to three times.

In summary, public health diseases adversely affect economic growth by exacerbating poverty and weakening human capital. However, the mechanistic analysis of existing studies is mostly focused on long-term epidemics, especially government financial aid, while mechanism of the impact of government epidemic prevention policy on economic growth under public health emergencies has not been sufficiently studied. Thus, it is impossible to form a set of economic countermeasures supported by scientific basis. In recent years, the frequent occurrence of public health emergencies had more rapidly and directly affected the lives of the population and economic production, and has increasingly become an uncontrollable factor in the process of economic growth in some countries. In view of this, this paper will take the COVID-19 as the background, combine the endogenous economic growth theory and epidemiological model, theoretically analyze the potential impact of government intervention policies on economic growth and its mechanism, and find the path of economic growth under the epidemic.

## Theoretical Model

Assume that *N*(*t*, τ) represents the current stock of infected cases at time *t* who have been infected at time τ. *N*(0, 0) = *N*_*i*_ is a constant. when *t* < τ, *N*(*t*, τ) = 0. Then, the total stock of cases at time *t* can be expressed as $N\left(t\right)=\int_{0}^{\infty }N\left(t,\tau \right)d\tau$. The spread of infectious diseases and government policy interventions are all related to variable *N*(*t*, τ). Assume that for government departments, the standard for effective control of infectious diseases is that the current stock Λ(*t*) at time *t* drops to 0, that is, Λ(*t*) = *N*(*t*, 0) → 0. We set an infectious factor β(τ) to represent the infectivity at time τ after been infected. Then the current case stock and the dynamic evolution process of the disease from time 0 to time *t* can be expressed as:


(1) $$\begin{array}{l} N\left(t,0\right)=\int_{0}^{t}\beta \left(\tau \right)N\left(t,\tau \right)d\tau \end{array}$$

(2) $$\begin{array}{l} \frac{\partial N\left(t,\tau \right)}{\partial t}+\frac{\partial N\left(t,\tau \right)}{\partial \tau }=0 \end{array}$$

### Intergenerational Transmission and Time Interval of the Virus

During the spread of the virus, one infected person usually infects more than one healthy person, forming intergenerational transmission. Most infectious viruses, including COVID-19, are transmitted between generations. The first generation of infected people tend to be those who come into close contact with the pathogen and become infected. While the second generation is infected by the first, and so on. In infectious diseases, as the chain of infection extends, the infectivity of the virus will gradually increase, and even the emergence of “super spreaders” means that the difficulty of prevention and control will be further increased. In view of this, we set an indicator *R*_0_ to measure the transmission capacity of the virus. Its epidemiological meaning is how many people can be transmitted by an infected person without external force. Obviously *R*_0_ can be obtained by β(τ) integral:


(3) $$\begin{array}{l} R_{0}=\int_{0}^{\infty }\beta \left(\tau \right)d\tau \end{array}$$

Another indicator of infectivity is the time interval of intergenerational transmission. That is, the average time interval from one infected person to another being infected by it. On the one hand, the more infectious, the shorter the time interval. On the other hand, the more effective and timely the government's epidemic prevention measures, the time interval of intergenerational transmission will be lengthened or even blocked. We define an intergenerational transmission time interval *T*_*g*_ which measured by the average of the infectious distribution:


(4) $$\begin{array}{l} T_{g}=\int_{0}^{\infty }\tau \beta \left(\tau \right)d\tau /\int_{0}^{\infty }\beta \left(\tau \right)d\tau \end{array}$$

Further, to facilitate the calculation of the model, we assume that the spread and attenuation of the virus are exponential growth or exponential decline, respectively. Based on this assumption, we concretize *N*(*t*, τ) into *N*(*t*, τ) = *e*^*rt*^. When *r* > 0, it means the spread of the disease, which also means that the infectious power is increasing (*R*_0_ > 1); when *r* < 0, it means that the infectious power is decreasing, corresponding to *R* < 1.

### Government Intervention: Isolate Existing Patients and Trace Close Contacts

*R*_0_ and *T*_*g*_ indicate the severity of the epidemic, which determines that the government needs to take intervention measures to prevent the epidemic from worsening. In order to control the spread of the epidemic, the strength and speed of relevant government measures will play an important role. In terms of response to COVID-19 and other sudden outbreaks, there are two main measures. One is to identify and publicize the clinical manifestations of the disease in a timely manner, and isolate those with symptoms promptly, and the other is to track and isolate close contacts to reduce intergenerational transmission.

#### Isolate the Sick Patients

Assuming that all people are susceptible to infection, *S*(τ) proportion of the infected people are in the incubation period (No clinical symptoms have yet been diagnosed). Although these potential patients will eventually become infected, that is, *S*(τ) tends to 0, but these patients will affect the effectiveness of isolation measures in the short term. Define ε_*I*_ as the strength of isolation measures, which can be measured by the proportion of patients being isolated. The larger the ε_*I*_ is, the more effective the isolation measures are. Through isolation, the infectiousness β(τ) will be reduced to β(τ)[1 − ε_*I*_(1 − *S*(τ))], and the patient stock at time *t* becomes:


(5) $$\begin{array}{l} N\left(t,0\right)=\int_{0}^{t}\beta \left(\tau \right)\left[1-\varepsilon_{I}\left(1-S\left(\tau \right)\right)\right]N\left(t,\tau \right)d\tau \end{array}$$

Through isolation, the intergenerational spread of diseases will tend to decrease. Defining *R*_*I*_ as the level of intergenerational transmission under isolation measures, there are:


(6) $$\begin{array}{l} R_{I}=\int_{0}^{\infty }\beta \left(\tau \right)\left[1-\varepsilon_{I}\left(1-S\left(\tau \right)\right)\right]d\tau \end{array}$$

For highly infectious diseases such as COVID-19, an infected person is contagious during the incubation period. So we define θ as the infectivity of the virus during the incubation period, $\theta =\int_{0}^{\infty }\beta \left(\tau \right)S\left(\tau \right)d\tau /\int_{0}^{\infty }\beta \left(\tau \right)d\tau$. Further, *R*_*I*_ = *R*_*o*_[1 − ε_*I*_ + ε_*I*_θ] can be obtained. Based on the foregoing assumptions, if the government takes strong isolation measures (ε_*I*_ = 1), the disease will gradually decline exponentially (*R*_*I*_ < 1), and then there will be θ < 1/*R*_*o*_. This shows that even under perfect isolate measures, the possibility of asymptomatic transmission still exists, which further shows that the government needs to track and isolate contagious close contacts.

#### Trace and Isolate Close Contacts

If the government takes measures to track close contacts, those who were infected at τ moment but did not become ill will be isolated. At this time, the instantaneous rate of change of *S*(τ) can be expressed as $h\left(\tau \right)=-\frac{1}{S\left(\tau \right)}\frac{dS\left(\tau \right)}{d\tau }$. To describe the infection process of close contacts, we expand *N*(*t*, τ) to *N*(*t*, τ, τ′), which represents the stock of people at time t infected at time τ by the patient who are infected at time τ′, that is, the patient at time τ is a close contact of the patient at time τ′. At the same time, defineε_*T*_as the strength of tracing close contacts, and *I*(*t*, τ, τ′) as the number of people infected but not isolated, and the meaning of “*t*, τ, τ′” is the same as the above content.

Further, we decompose the *I*(*t*, τ, τ′) group into four parts: Part I, population will not be isolated and traced is denoted as ($I_{Ī\bar{T}}$); Part II, population chooses self-isolate but will not be traced is denoted as ($I_{I\bar{T}}$); Part III, population will not be isolated but will be traced is denoted as (*I*_Ī*T*_); Part IV, population either will be isolated or will be traced is denoted as (*I*_*IT*_). Define the differential operator as $\Delta \;=\;\partial_{t}+\partial_{\tau }+\partial_{\tau^{\prime }}$, then we have $\Delta I_{Ī\bar{T}}=0$, $\Delta I_{I\bar{T}}=-h\left(\tau \right)I_{I\bar{T}}$, $\Delta I_{ĪT}=-h\left(\tau^{\prime }\right)I_{ĪT}$, $\Delta I_{IT}=-\left[h\left(\tau \right)+h\left(\tau^{\prime }\right)\right]I_{IT}$. We add up the patients at time τ′ to calculate the number of patients in this group at time τ to reach the stock Λ(*t*, τ) at time t, which is $\Lambda \left(t,\tau \right)=\beta \left(\tau \right)\int_{\tau }^{t}I\left(t,\tau ,\tau^{\prime }\right)d\tau^{\prime }$. Based on this, the policy effect of tracing and isolation can be expressed as:


(7) $$\begin{array}{l} I_{I\bar{T}}\left(t,\tau \right)=\left(1-\varepsilon_{I}\right)\left(1-\varepsilon_{T}\right)\Lambda \left(t,\tau \right) \end{array}$$

(8) $$\begin{array}{l} I_{I\bar{T}}\left(t,\tau \right)=\varepsilon_{I}\left(1-\varepsilon_{T}\right)\Lambda \left(t,\tau \right) \end{array}$$

(9) $$\begin{array}{l} I_{ĪT}\left(t,\tau \right)=\varepsilon_{T}\left(1-\varepsilon_{I}\right)\Lambda \left(t,\tau \right) \end{array}$$

(10) $$\begin{array}{l} I_{IT}\left(t,\tau \right)=\varepsilon_{I}\varepsilon_{T}\Lambda \left(t,\tau \right) \end{array}$$

Take *I*_*IT*_(*t*, τ) as an example. In an ideal situation, if the government's tracing and isolation policies are fully strong (ε_*T*_ = 1 and ε_*I*_ = 1), all potential patients will be tracked, namely *I*_*IT*_(*t*, τ) = Λ(*t*, τ). However, not all policies can achieve the desired results, and varying degrees of policy friction loss will reduce the policy effect.

Under the “tracing + isolation” policy, the dynamic changes of *N*(*t*, τ, τ′) can be represented by *I*(*t*, τ, τ′):


(11) $$\begin{array}{l} I_{Ī\bar{T}}\left(t,\tau ,\tau^{\prime }\right)=N_{Ī\bar{T}}\left(t,\tau ,\tau^{\prime }\right) \end{array}$$

(12) $$\begin{array}{l} I_{I\bar{T}}\left(t,\tau ,\tau^{\prime }\right)=\exp\left[-\int_{0}^{\tau }h\left(u\right)du\right]\\ N_{I\bar{T}}\left(t,\tau ,\tau^{\prime }\right)=S\left(\tau \right)N_{I\bar{T}}\left(t,\tau ,\tau^{\prime }\right) \end{array}$$

(13) $$\begin{array}{l} I_{ĪT}\left(t,\tau ,\tau^{\prime }\right)=\exp\left[-\int_{\tau^{\prime }-\tau }^{\tau^{\prime }}h\left(u\right)du\right]\\ N_{ĪT}\left(t,\tau ,\tau^{\prime }\right)=\frac{S\left(\tau^{\prime }\right)}{S\left(\tau^{\prime }-\tau \right)}N_{ĪT}\left(t,\tau ,\tau^{\prime }\right) \end{array}$$

(14) $$\begin{array}{l} I_{IT}\left(t,\tau ,\tau^{\prime }\right)=\exp\left[-\int_{0}^{\tau }h\left(u\right)du\right]\exp\left[-\int_{\tau^{\prime }-\tau }^{\tau^{\prime }}h\left(v\right)dv\right]\\ N_{IT}\left(t,\tau ,\tau^{\prime }\right)=\frac{S\left(\tau \right)S\left(\tau^{\prime }\right)}{S\left(\tau^{\prime }-\tau \right)}N_{IT}\left(t,\tau ,\tau^{\prime }\right) \end{array}$$

Because of $I=I_{Ī\bar{T}}+I_{ĪT}+I_{I\bar{T}}+I_{IT}$, we can substitute the above formula into $\Lambda \left(t,\tau \right)=\beta \left(\tau \right)\int_{\tau }^{t}I\left(t,\tau ,\tau^{\prime }\right)d\tau^{\prime }$ and simplify it. Then the effect of the intervention policy can be obtained:


(15) $$\begin{array}{l} \Lambda \left(t,\tau \right)=\beta \left(\tau \right)\left[1-\varepsilon_{I}\left(1-S\left(\tau \right)\right)\right]\\ \int_{\tau }^{t}N\left(t,\tau ,\tau^{\prime }\right)\left(1-\varepsilon_{T}+\varepsilon_{T}\frac{S\left(\tau^{\prime }\right)}{S\left(\tau^{\prime }-\tau \right)}\right)d\tau^{\prime } \end{array}$$

The above models are consistent with Fraser et al. (29). On this basis, we further expand to a model of economic growth under infectious diseases and government intervention.

### The Intervention Measures and Economic Growth

Based on the above assumptions, all patients in the incubation period will become ill and need to be isolated, treated and recovered. Then we further assume that the infected people will not be able to provide labor during the period of the epidemic.

#### The Epidemic Damages Labor

Based on the government's isolation and tracing measures, the epidemic will affect economic production by weakening the number and quality of labor. Suppose the production function at this time is:


(16) $$\begin{array}{l} Y=\;\varepsilon_{I}\varepsilon_{T}F\left(K,A\left(L-N\right)\right) \end{array}$$

Where *A* is the labor efficiency of the Solow-Swan model, *L* − *N* is the actual labor input in the production process, and. Based on the above model, we can obtain the patient stock at time *t* through time accumulation. In the Solow-Swan model, the time factor enters the production function through *K, L*, and *A* in an indirect way. Therefore, to simplify the model, we only consider the output in period *t*, and the subscript *t* is removed. The formula (16) shows the impact of the epidemic on production from two aspects. On the one hand, patients isolated due to the epidemic will not be able to work, and only *L* − *N* proportion of the labor force will participate in production. On the other hand, the potentially infected population indirectly affects the economic production process through their own disease and infects others. In order to simplify the model, we replace this shock with the government's isolation efforts ε_*I*_ and tracing efforts ε_*T*_. The more efficiency of the government policies, the smaller the impact on economic output may be. For labor force *L*, we set the process as the difference between untracked asymptomatic patient and death in the workforce:


(17) $$\begin{array}{l} \frac{dL}{dt}=\theta \left(1-\varepsilon_{T}\right)L-\phi N \end{array}$$

Where θ is the infectivity of the virus during the incubation period, θ(1 − ε_*T*_)*L* is the number of potential infections but not tracked in the labor force, and ϕ is the case fatality rate.

#### The Epidemic Damages Production Capacity

In addition to directly affecting the health of the labor force, the epidemic has also caused enterprises to stop production and slow production efficiency to restart. Especially for the majority of small and medium-sized enterprises, the delay in resuming work will comprehensively affect their production efficiency through capital flow and labor. Therefore, we assume that during the epidemic, the change in total factor productivity obeys the following process:


(18) $$\begin{array}{l} \frac{dA}{dt}=\upsilon \left(A^{*}-A\right) \end{array}$$

Where A^*^ is the steady-state level of total factor productivity, and υ is the recovery efficiency of economic productivity. During the epidemic and for a period of time afterwards, the government needs to use fiscal policy, monetary policy and other means to repair the damaged economy. For simplicity, we abstract the above policy into a government subsidy *R* and denote it as *R* = ζY. Then the recovery efficiency of production capacity υ is closely related to the above-mentioned government measures. Assuming that the government subsidizes production capacity based on the unit labor output at level A^*^, then the relationship between υ and *R* can be expressed as:


(19) $$\begin{array}{l} \upsilon =\frac{\pi R}{A^{*}L} \end{array}$$

Where π represents the proportion of government subsidies for restoring production capacity.

### Equilibrium of the Model

The equilibrium of the model is realized in two parts. One is that the government controls the epidemic situation by isolating and tracing close contacts; the other is that, through policy assistance, economic production is restored to the effective frontier level before the epidemic.

#### The Balance of the Epidemic

Regarding the epidemic, the government will adopt a series of measures so that the new infections will eventually reach zero. Therefore, the equilibrium condition of the model can be expressed as:


(20) $$\begin{array}{l} \Delta N\left(t,\tau ,\tau^{\prime }\right)\;=\;0 \end{array}$$

Then, *N*(*t*, 0, τ) = Λ(*t*, τ). Available differential operators:


(21) $$\begin{array}{l} \beta \left(\tau \right)\left[1-\varepsilon_{I}\left(1-S\left(\tau \right)\right)\right]\int_{0}^{\infty }\left(1-\varepsilon_{T}+\varepsilon_{T}\frac{S\left(\rho +\tau \right)}{S\left(\rho \right)}\right)\left(▪\right)d\rho \end{array}$$

If close contacts are screened and traced before symptoms appear, then tracing close contacts may be considered a recursive process. Until all infected contacts are isolated, then the eigenvalue of the differential operator tends to 1. And the eigenvector of the epidemic growth and decline threshold is consistent with the steady-state distribution of the infection time. It can be assumed that the number of patients in the incubation period is exponentially distributed *S*(τ) = *e*^−*vτ*^, then the above formula can be simplified to:


(22) $$\begin{array}{l} \int_{0}^{\infty }\beta \left(\tau \right)\left[1-\varepsilon_{I}\left(1-S\left(\tau \right)\right)\right]\left(1-\varepsilon_{T}+\varepsilon_{T}S\left(\tau \right)\right)d\tau =1 \end{array}$$

Further assuming $\beta \left(\tau \right)\;=\;R_{o}e^{-\tau }$, then $\theta =\int_{0}^{\infty }\beta \left(\tau \right)S\left(\tau \right)d\tau /\int_{0}^{\infty }\beta \left(\tau \right)d\tau =1/\left(v+1\right)$. Simplifying equation (22), the steady state of the epidemic can be obtained as:


(23) $$\begin{array}{l} R_{o}\{\left[\left(1-\varepsilon_{I}\right)\left(1-\varepsilon_{T}\right)+\varepsilon_{I}\left(1-\varepsilon_{T}\right)\right]\theta \\ +\;\left(1-\varepsilon_{I}\right)\varepsilon_{T}\theta +\varepsilon_{I}\varepsilon_{T}\frac{\theta }{2+\theta }\}=1 \end{array}$$

Where θ measures the infectivity of the virus under asymptomatic conditions. It is related to the incubation period of the virus itself, so it is relatively stable and regarded as a fixed parameter. Then (23) should show the relationship between the virus infectivity *R*_*o*_ under steady-state conditions and different policy efforts (ε_*I*_, ε_*T*_). we can then realize the simulation analysis of *R*_*o*_ through the adjustment of (ε_*I*_, ε_*T*_).

#### Equilibrium of Economy

We assume that the process of capital accumulation is:


(24) $$\begin{array}{l} \dot{K}=\sigma Y-\delta K \end{array}$$

Where σ is the savings rate and δ is the capital depreciation rate. Let *y* = *F*(*k*, 1), where *k* ≡ *K*/*A*(*L* − *N*) is the effective per capita capital, then the effective per capita capital differential equation of the Solow-Swan model can be expressed as:


(25) $$\begin{array}{l} \dot{k}=\sigma \varepsilon_{T}\varepsilon_{I}y-\left(\delta +g+l\right)k \end{array}$$

In steady state, we can get σε_*T*_ε_*I*_*y* = (δ + *g*+*l*)*k*. In the classic Solow-Swan model, labor and technology are given directly exogenously. However, under the conditions of the epidemic, how much labor can participate in production and how much productivity is restored are all affected by the measures to control the epidemic. Therefore, *n* and *g* are related to the efficiency of isolation and tracing.

Let *g* ≡ (*dA*/*dt*)/*A* be the rate of technical recovery, and *a* = *A*/A^*^ be the relative production efficiency under the epidemic. From the equation (16)–(19), we get:


(26) $$\begin{array}{l} g=\pi \zeta \varepsilon_{T}\varepsilon_{I}\left(1-\beta \right)y\left(1-a\right) \end{array}$$

The labor growth rate can be derived from equation (17):


(27) $$\begin{array}{l} l\equiv \left(dL/dt\right)/L=\theta \left(1-\varepsilon_{T}\right)-\phi \beta \end{array}$$

Substituting (26) and (27) into ε_*I*_*y* = (δ + *g*+*l*)*k* can obtain the balanced growth path of the economy under epidemic intervention measures:


(28) $$\begin{array}{l} k=\sigma \varepsilon_{T}\varepsilon_{I}y/\left[\delta +\pi \zeta \varepsilon_{T}\varepsilon_{I}\left(1-\beta \right)y\left(1-a\right)+\theta \left(1-\varepsilon_{T}\right)-\phi \beta \right] \end{array}$$

Suppose the output function is $Y=\varepsilon_{I}\varepsilon_{T}K^{\alpha }{\left[A\left(L-N\right)\right]}^{1-\alpha }$, then:


(29) $$\begin{array}{l} y=\frac{Y}{A\left(L-N\right)}=\varepsilon_{I}\varepsilon_{T}{\left(\frac{K}{A\left(L-N\right)}\right)}^{\alpha }=\varepsilon_{I}\varepsilon_{T}k^{\alpha } \end{array}$$

Through the two formulas (28) and (29), it can be find that both the path of per capita capital growth and the path of economic growth are closely related to the government's policy to control the epidemic. Based on this, according to different combinations of (ε_*I*_, ε_*T*_) policies, images of *R*_*o*_, *k*, and *y* can be drawn, so that the equilibrium process of the infection rate and the recovery of economic output can be observed.

## Parameter Settings

The variables simulated and analyzed in this paper are the relationship between the virus transmission power *R*_0_, the per capita capital stock *k*, and the per capita output *y*, and the government's policy strength (ε_*I*_, ε_*T*_) for isolating and tracing close contacts. The paths of virus transmissibility *R*_0_, capital stock per capita *k* and output per capita *y* were observed under different policy combinations.

For the infectious index *R*_*o*_ of the COVID-19, the WHO estimates that its infectivity fluctuates between 1.4 and 2.5, which is equivalent to influenza. Since the epidemic situation has not yet been fully resolved, the estimate of *R*_*o*_ given by the academic community based on different research methods and survey samples is only a reference (30–32). In fact, *R*_*o*_ is closely related to the policies adopted by the government. The prevention and control measures of the epidemic in different countries are very different. So it is difficult to accurately estimate an accurate data interval.

We use the data of China in January to February 2020 (when the epidemic is severe). In terms of epidemic prevention and economic restoration, China has given more efficient policy arrangements. Regarding the isolation policy ε_*I*_, Chinese government has adopted multiple measures such as centralized isolation of the affected population, home isolation of healthy people, and traffic control, which brought the epidemic to an inflection point in a short period of time. Regarding the tracing policy ε_*T*_, China has improved the efficiency of screening suspected cases through full coverage of nucleic acid testing.

For other infectious disease parameters, Yang et al. (28) predicted the development trajectory of the COVID-19 in China, and estimated the infectivity coefficient β and the fatality rate ϕ. We refer to the results of Yang et al. (28) and set the infectivity coefficient β and case fatality rate ϕ to 0.157 and 0.03, respectively. For the asymptomatic infection rate θ, its meaning in this article is the infectivity of the incubation period. In the parameter estimation of Yang et al. (28), the estimated value of the incubation period reproduction rate is 7, and we use this value to approximate θ.

For government expenditures during the epidemic, we estimate based on real data. ζ is the proportion of fiscal-related expenditures to GDP during the period of the epidemic. According to data disclosed on the website of the State Council of China, during January to February 2020, China has invested 116.9 billion yuan in epidemic prevention, and 103 billion yuan to ensure the basic livelihood. Regarding GDP, we estimate the GDP from January to February 2020 based on industrial value added data. According to the National Bureau of Statistics of China, the value added of industrial enterprises above designated size from January to February 2020 actually dropped by 13.5%, which approximates the GDP volatility of the same period. According to the GDP in the first quarter of 2019, the GDP of the first quarter of 2019 is 21.3433 trillion yuan. Normally, in the first quarter of GDP, January-February accounted for 60% and March accounted for 40%. We estimate that the GDP from January to February 2020 is about 11,077.2 billion yuan. Thus, ζ is ~0.02.

The subsidy efficiency π of fiscal expenditures to production capacity represents the proportion of epidemic-related fiscal expenditures used to restore production capacity. During the epidemic, enterprises were greatly impacted. According to data disclosed by the Ministry of Finance of PRC, for small-scale taxpayers who are severely affected by the epidemic and whose annual sales are <5 million yuan, the tax rate will be 1%, and those whose annual sales are <100,000 yuan will be exempt. In view of the capital difficulties faced by some enterprises that have resumed work and production, a discount interest rate of 50% of the actual loan interest rate will be given to the enterprises. These measures covered more than 80% of tax payers, thereby helping them to resume production. Based on this, we define the efficiency of government capacity recovery subsidies as 0.8.

Regarding the relative production efficiency *a* under the epidemic, we measure it by the level of production recovery. Electricity consumption has the most say in the extent of industrial capacity recovery. Therefore, according to the data disclosed by the National Development and Reform Commission of China, in the first 2 months of 2020, the electricity consumption of the whole society fell by 7.8%. Electricity decreased by 12.0%. This data is consistent with the actual decline of 13.5% in the added value of industrial enterprises from January to February 2020 disclosed by the National Bureau of Statistics of China. Therefore, we set the relative production efficiency during the epidemic to 0.88 on the basis of a 12% decline in electricity consumption in the secondary industry. For the capital depreciation rate and savings rate, set to 0.1 and 0.45, respectively.

## The Effects of Policy and the Path of Economic Growth

In the absence of control over public health emergencies, the infectiousness of diseases, per capita capital, and per capita output will become uncontrollable. The contagiousness of the disease depends on the ability of the virus to spread and the mobility of the population, while the per capita capital and per capita output depend on the repair power of the economy itself. However, under the circumstance that the government actively intervenes, things may turn for the better. We use the data of the COVID-19 as a benchmark to simulate the relationship between the virus transmission capacity *R*_0_, per capita capital *k*, and economic growth path *y*, and policy (ε_*I*_, ε_*T*_), so as to find the changing path of the virus transmission capacity and the growth path of per capita capital and economic growth path under different intensity of government intervention policies. Then, we change the intensity of β to simulate the changes in the path of economic growth under different infectious public health events.

### The Effect of Policy and the Economic Path Under the COVID-19

**Figure 1:**
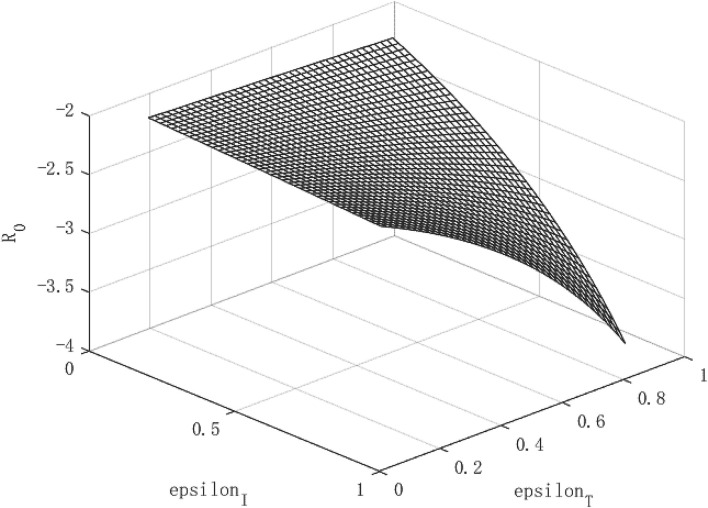
Virus transmission path of COVID-19.

**Figure 2:**
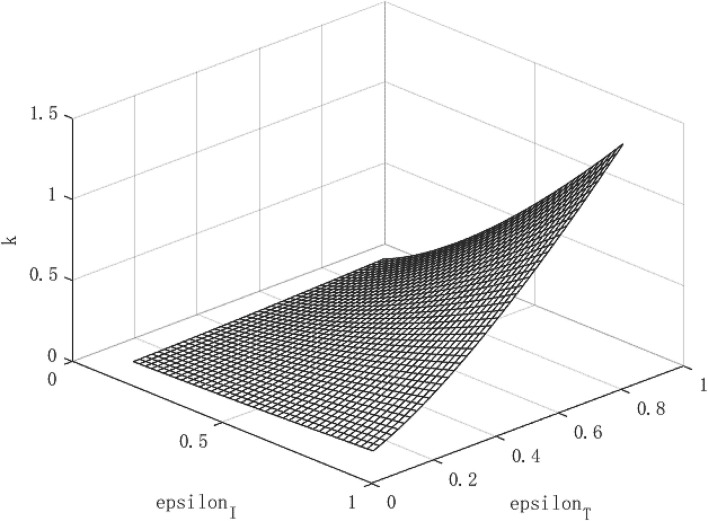
Growth path of capital per capita under COVID-19.

**Figure 3:**
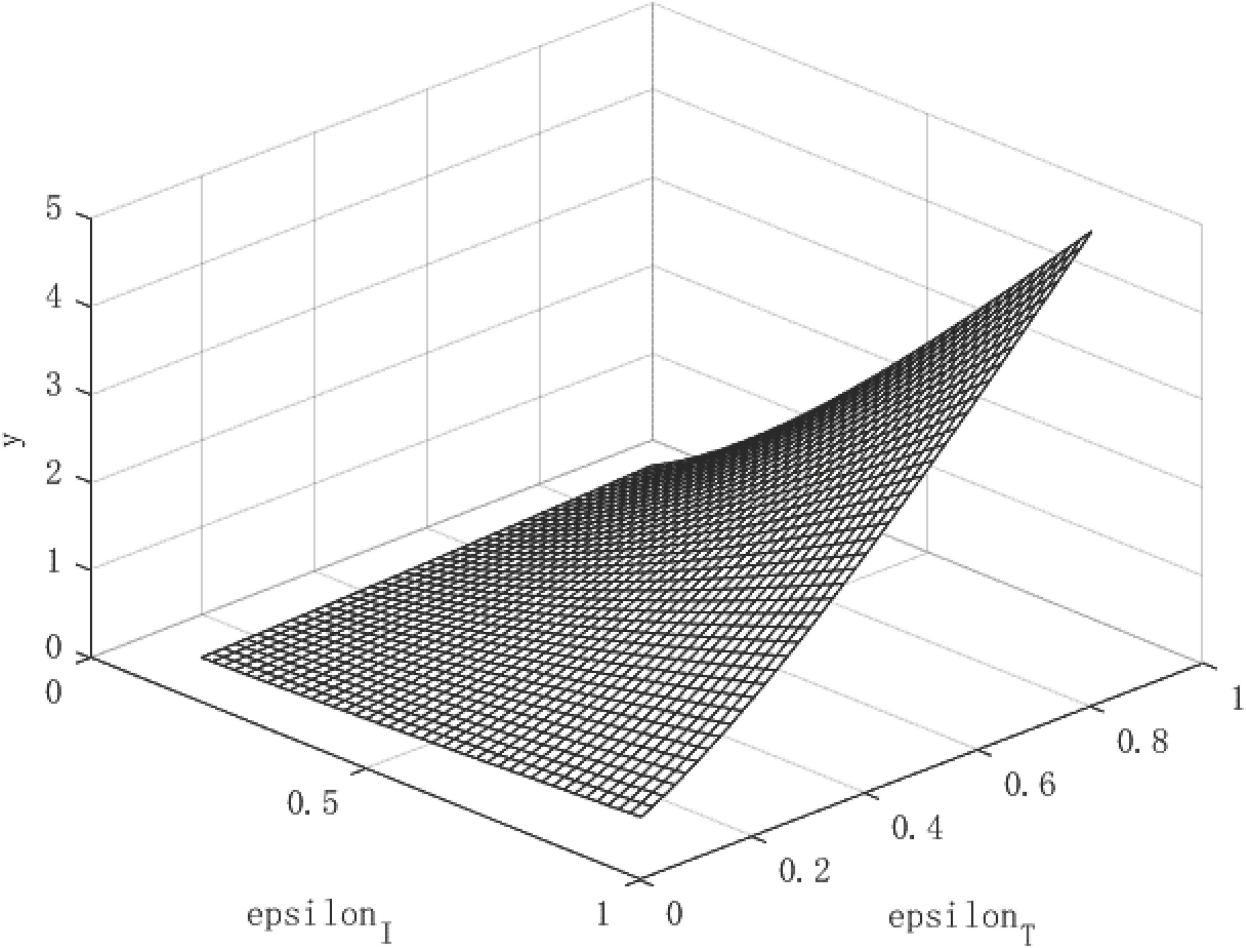
Growth path of per capita output under COVID-19.

Figures 1–**3** show the effect of government intervention and the path of economic growth under the impact of the COVID-19. It can be seen from Figure 1 that both isolation and tracing close contacts will reduce the infectivity of the virus. Comparing isolation and tracing measures, it can be found that tracing close contacts can reduce the spread of the virus by up to 4%, while isolation measures can only reduce the spread of the virus by about 3%. This shows that measures to track close contacts have a greater impact on reducing the spread of the virus than of isolation. This is mainly because the isolation measures only isolate symptomatic infections from the normal population, and those asymptomatic infections are still hidden in the normal population, which is extremely detrimental to reducing the spread of the virus. For the tracing measures, through population screening, asymptomatic infected people can be found and isolated, which will greatly reduce the probability of healthy people being exposed to the virus, so it has a more positive effect on reducing the spread of the virus. This also verified the fact that China minimized the spread of the virus within 6 months after the outbreak of the COVID-19 and the economy quickly recovered.

Figure 2 shows the growth path of per capita capital under different policy combinations. It can be found that the isolation measures and tracing measures adopted by the government will have a positive impact on per capita capital. However, comparing the two policy measures, it can be found that tracing close contacts have a better impact on the capital stock per capita than the isolation measures. The tracing measures can increase the per capita capital stock by up to about 1.3%, while the isolation measures can increase the per capita capital stock by up to 0.3%. Figure 3 shows the growth path of per capita output. It can be found that although the epidemic restricts labor and production capacity, under the government intervention policy, it has a positive effect on output. This is manifested in that both isolation and tracing measures will increase the level of output per capita. However, the isolation measures can increase per capita output by about 0.5%, while the chasing policy can increase per capita output by about 4.5%. The effect of tracing is better than that of isolation measures. This is because tracing measures can find potential patients affected by the virus and prevent more healthy people from being infected, thereby ensuring a continuous supply of healthy labor for economic production, which is of great significance for the rapid recovery of the economy.

Although the effect of tracing measures is better than isolation measures, the use of isolation measures cannot be abandoned. Because isolation measures are the prerequisite for tracing measures, the tracked patients need to be effectively isolated for the policy to play a substantial role. Under the combination of the two policies, the path of economic growth takes on an upward parabolic shape.

### The Effects of Policy Intervention and the Path of Economic Growth Under the Shack of a Low Contagious Public Health Emergencies

**Figure 4:**
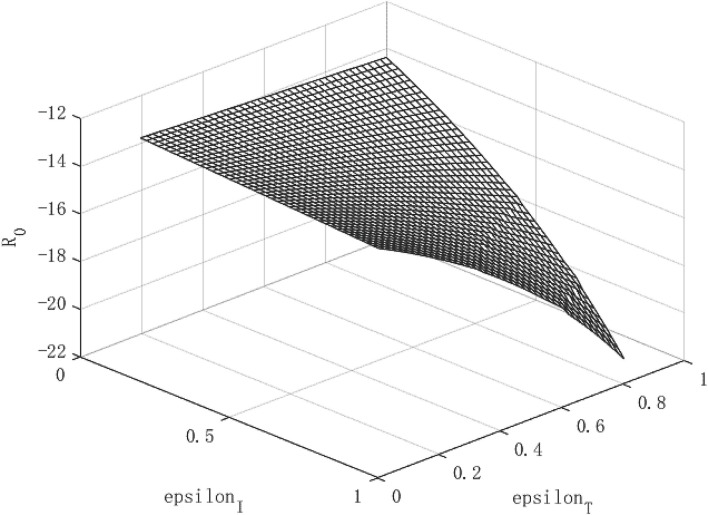
Virus Transmission Path of low contagious public health emergencies.

**Figure 5:**
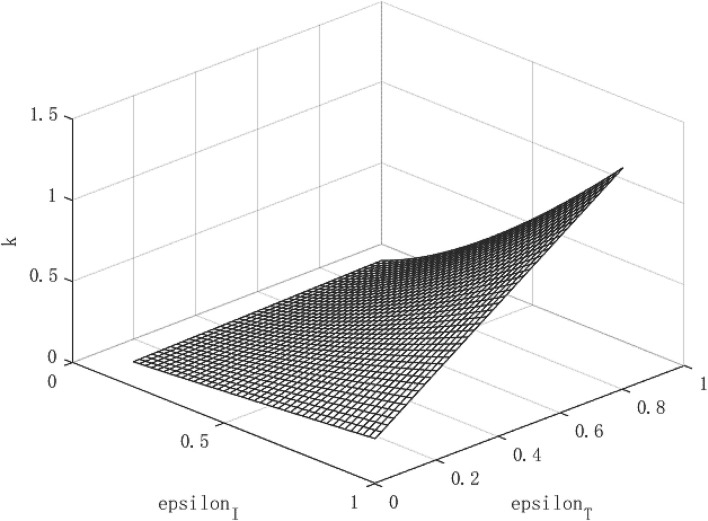
Growth path of per capita capital under low contagious public health emergencies.

**Figure 6:**
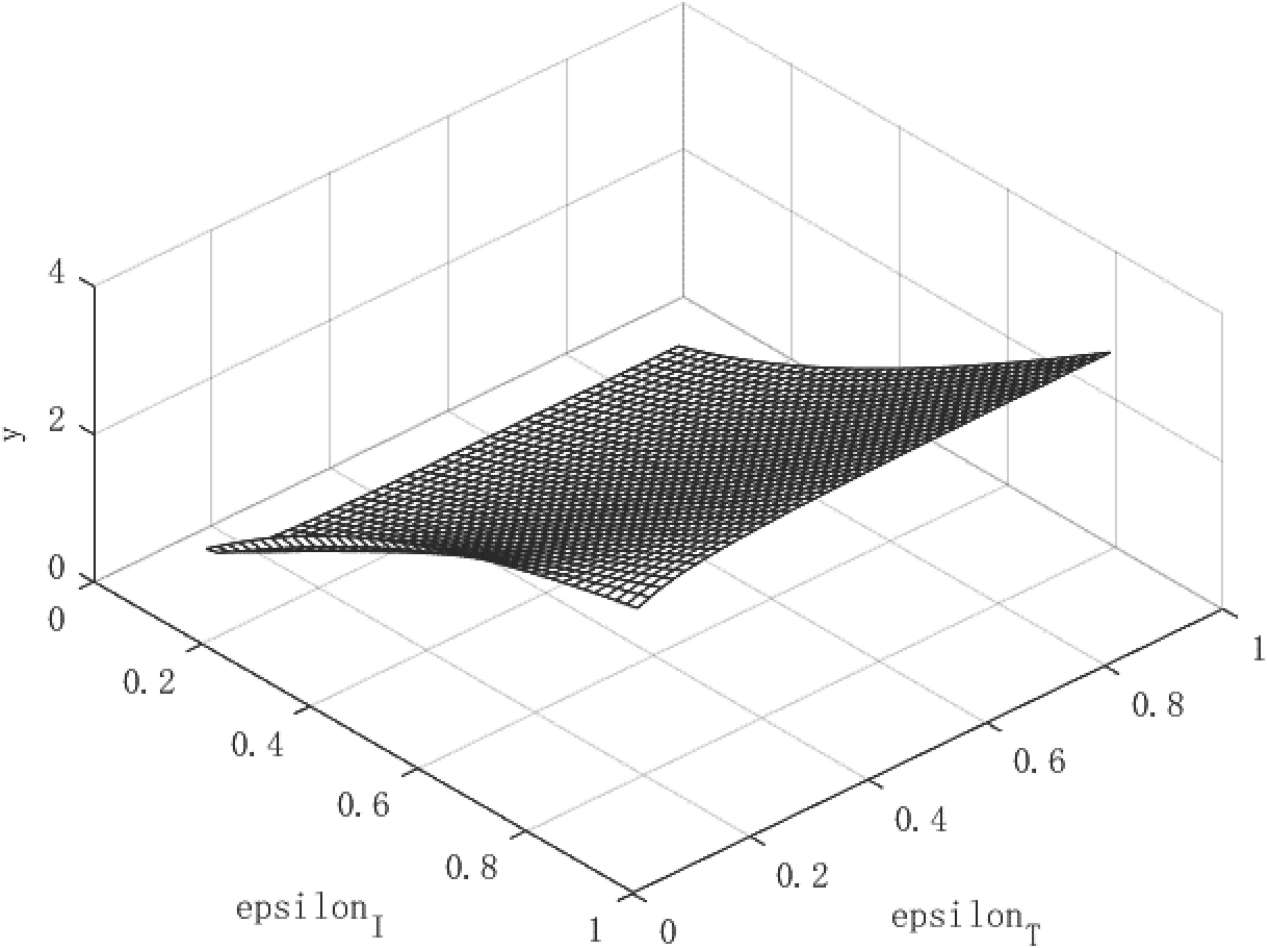
Growth path of per capita output under low contagious public health emergencies.

Figures 4–**6** show the policy effects and economic growth path under a weakly contagious epidemic. We define weak infectiousness as the lower infectious beta = 0.0785 relative to the infectiousness of COVID-19 (beta = 0.157). Analyzing Figure 4, we can find that the spread of the virus is negatively correlated with isolation measures. Because the virus is relatively weak, both isolation and tracing measures can significantly reduce the virus' infectivity. The isolation measures can reduce the infectivity of the virus by about 16%, while tracing measures can reduce the spread of the virus by about 21%. Overall, the effects of tracing measures is due to isolation measures.

Figure 5 shows the relationship between per capita capital stock and intervention policies. It can be found that there is a positive correlation between the per capita capital stock and the intensity of the isolation measures, and the isolation measures can increase the per capita capital stock by about 0.3% at most. At the same time, tracing measures are positively correlated with per capita capital stock in most cases, and it can increase the per capita capital existence by up to about 1.2%. Figure 6 shows the relationship between output per capita and intervention policies. It can be found that both isolation measures and tracing measures have an effect on per capita output, and both can increase per capita output by up to 3%.

On the whole, under a weakly contagious epidemic, tracing and isolation measures can increase the level of output per capita, but tracing measures are more obvious for the per capita capital stock and virus transmission. Therefore, for weakly infectious viruses, it is still necessary to track asymptomatic infections to prevent the spread of the epidemic from spreading to the economy.

### The Effects of Policy Intervention and the Path of Economic Growth Under the Shaock of a Highly Contagious Public Health Emergencies

**Figure 7:**
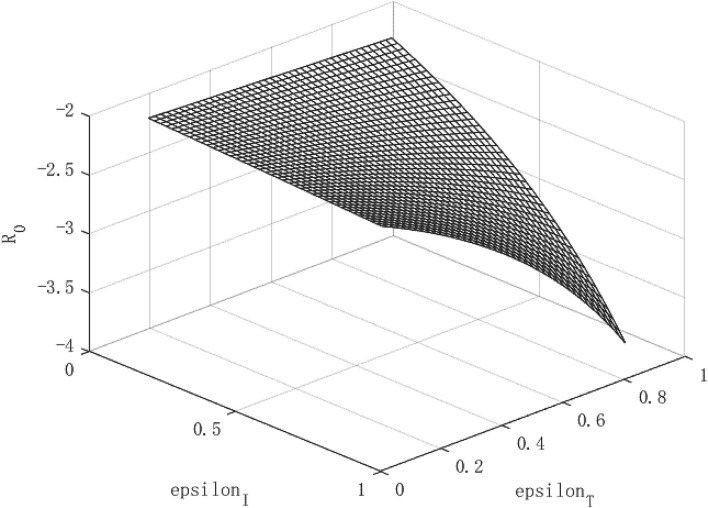
Virus Transmission Path of high contagious public health emergencies.

**Figure 8:**
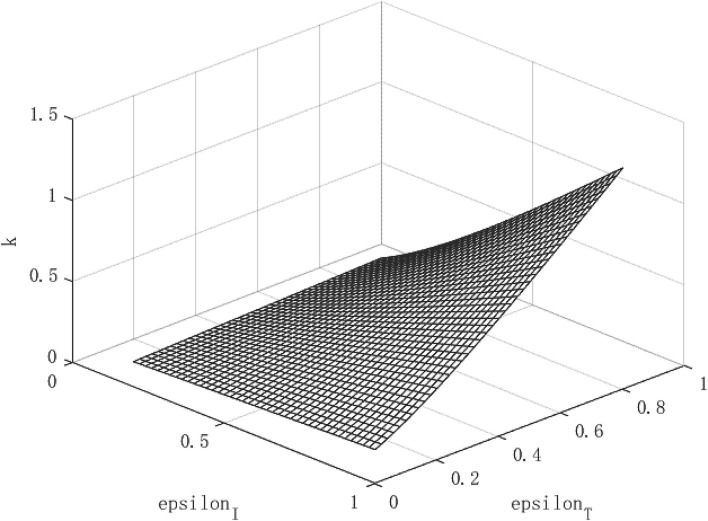
Growth path of per capita capital under high contagious public health emergencies.

**Figure 9:**
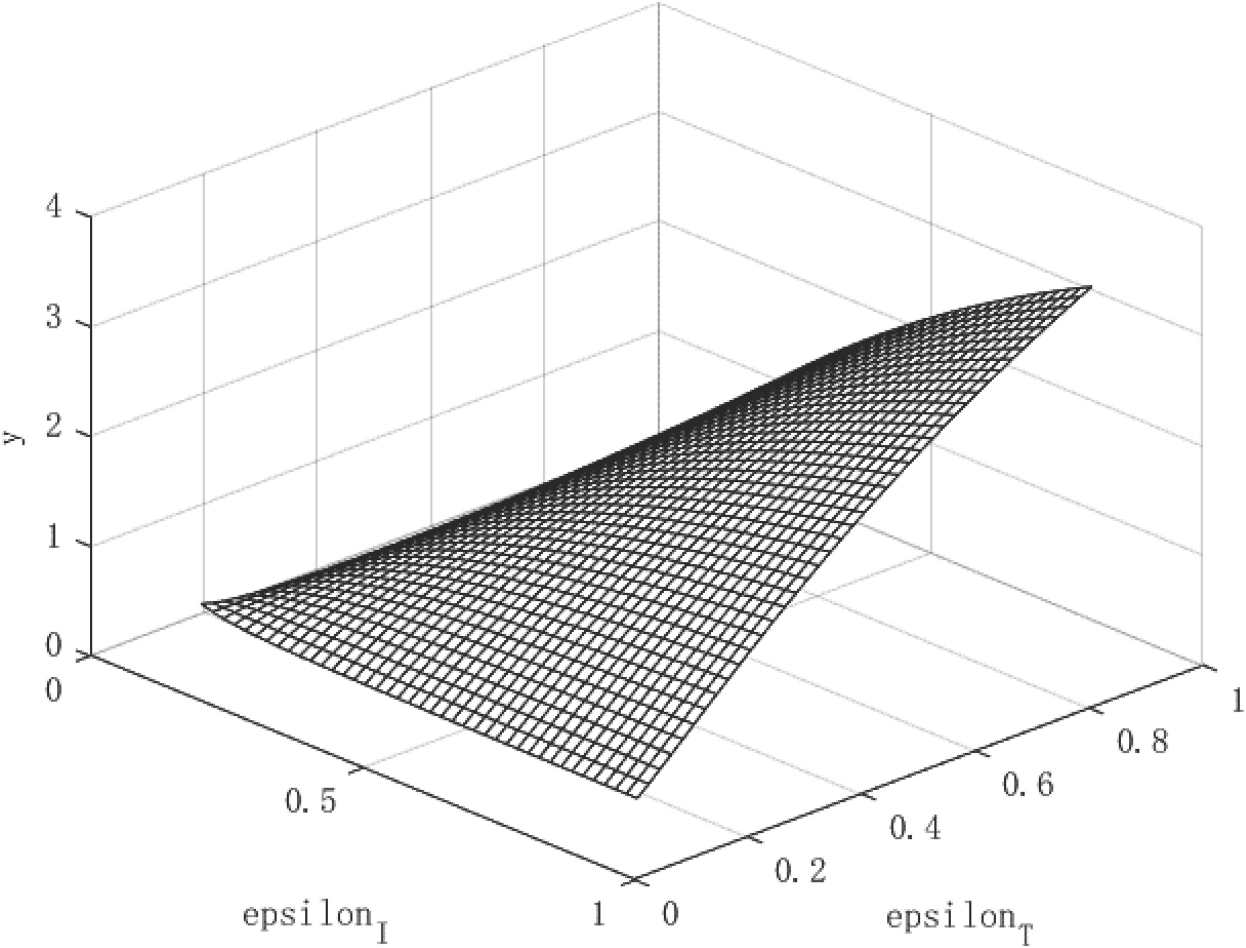
Growth path of per capita output under high contagious public health emergencies.

Figures 7–**9** show the effect of government policies and the path of economic growth when a virus that is more contagious than the COVID-19. We doubled the infectivity of the virus to beta = 0.38 for simulation. It can be seen from Figure 7 that when a highly contagious virus occurs, the isolation measures and tracing measures can still reduce the spread of the virus. The tracing measures are better than the isolation measures.

In terms of per capita capital stock and per capita output (Figures 8, 9), isolation measures and tracing measures have a positive impact on the above two, which does not change the previous conclusions. However, we found more differences in some details. The end of the growth path of per capita capital stock and economic output has become more gradual, which is different from the steeper curved end in Figures 1–3. This indicates that even if the government adopts the most stringent isolation and tracing measures for highly contagious viruses, the effect of repairing the negative economic impact of the epidemic is limited, and the attenuation of the policy is shown at the end of the curved surface.

### The Robust Test of the Model

**Figure 10:**
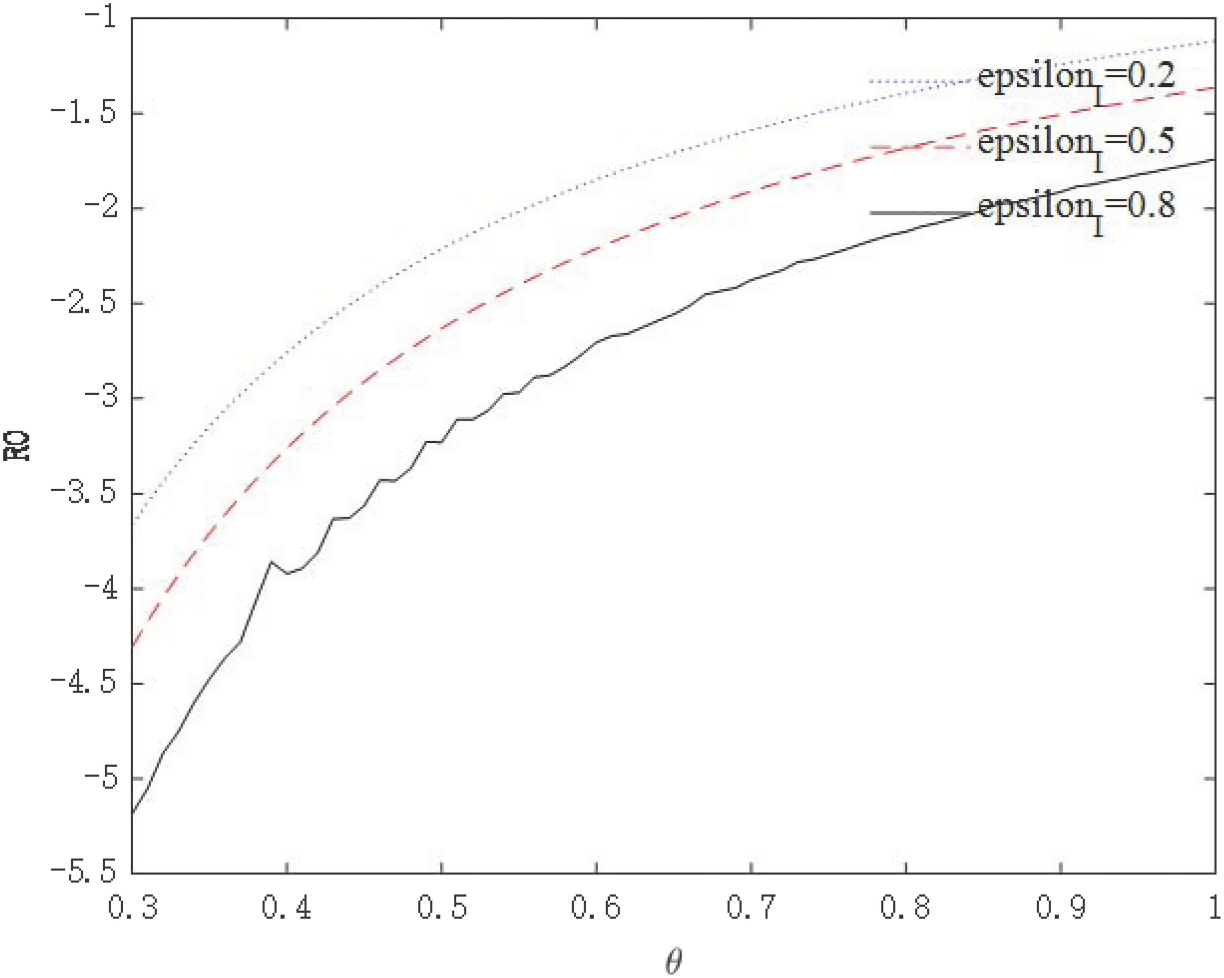
The relationship between *R*_0_ and θ under isolation measures.

**Figure 11:**
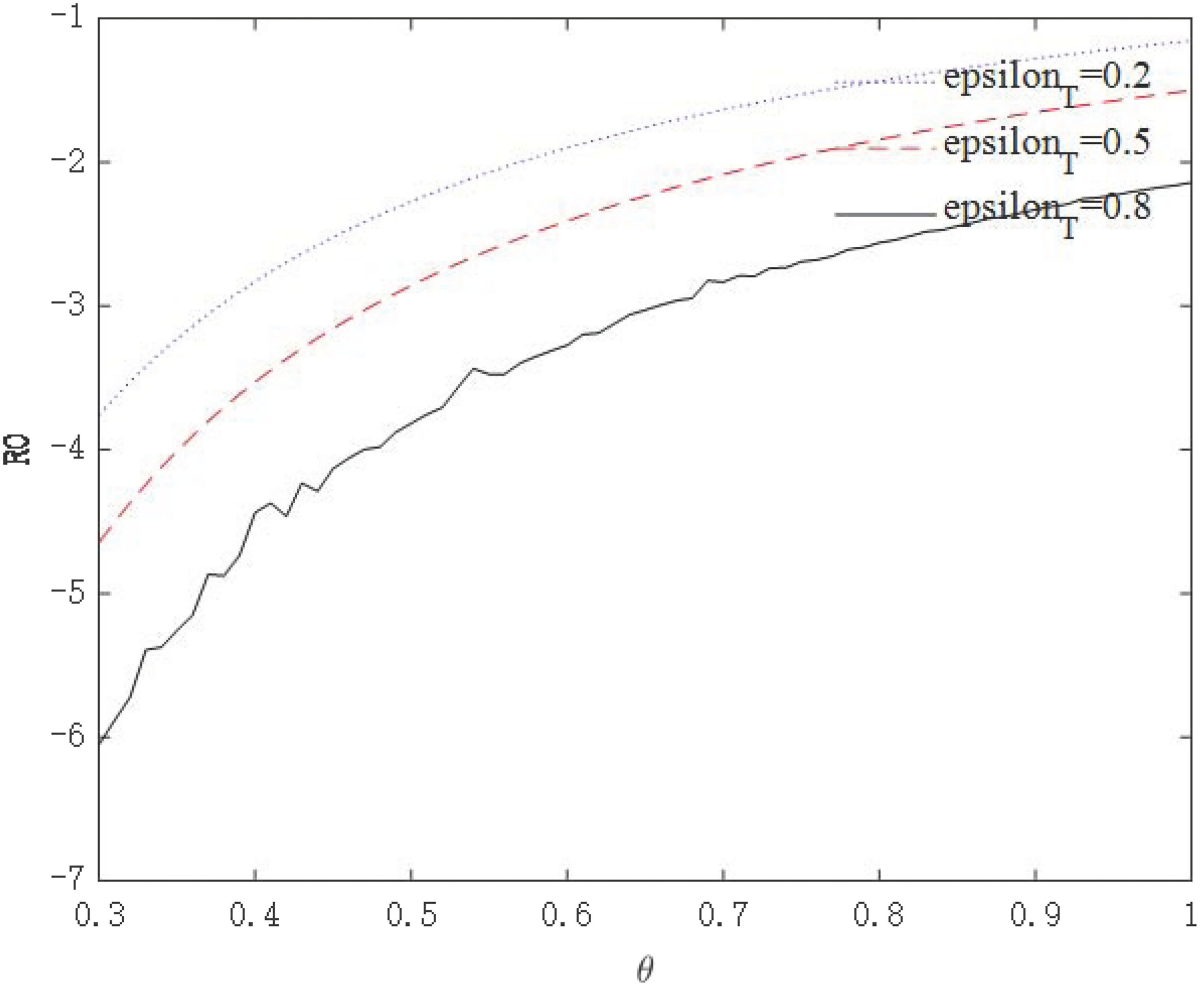
The relationship between *R*_0_ and θ under tracing measures.

The above analysis show that through government intervention, the infectious power of the virus will be weakened, but we seem to be unable to give an optimal policy combination plan through the path of economic growth. Because the purpose of these policies is to control the epidemic, followed by safeguarding the economy. Therefore, we need to further observe whether government interventions can affect the asymptomatic infection rate θ through *R*_0_. Figures 10, 11 shows the relationship between virus infection intensity *R*_0_ and asymptomatic infection rate θ under different policy strengths. We assign 0.2, 0.5, and 0.8 to ε_*I*_ and ε_*T*_ to represent three different policy intervention intensities. Figure 10 shows the relationship between *R*_0_ and θ under isolation measures, and Figure 11 shows the relationship between *R*_0_ and θ under tracing measures.

From the perspective of the relationship between policy intensity and *R*_0_, the greater the policy intensity, the more obvious the drop in *R*_0_. The negative impact of tracing measures on *R*_0_ is greater than that of isolation measures. From the perspective of the relationship between θ and *R*_0_, the larger θ is, the more infectious the virus is. As θ becomes larger, the effect of intervention policies on reducing *R*_0_ becomes weaker. For highly infectious viruses, the most stringent intervention measures should be taken to minimize the spread of the virus. These conclusions conform to the basic logic and prove that the model is robust.

## Research Conclusions and Policy Recommendations

We build a partial equilibrium model of economic growth on the basis of an infectious disease model, and simulates the impact of government intervention measures on economic growth. The results show that although the epidemic has restricted labor and production capacity, it has a positive effect on economic recovery under government intervention policies. Government intervention is non-linear to the spread of the epidemic and the path of economic growth. On the one hand, measures to track close contacts and isolation measures are conducive to reducing the spread of the virus, and contribute to the increase of per capita capital stock and per capita output, but the effects of tracing close contacts are better than the effects of isolation measures. On the other hand, for infectious diseases of different intensities, the economic growth path under government intervention policies is different. Under low infectious public health emergencies, isolation and tracing measures can increase per capita output. Considering that the spread of the virus and per capita capital stock are more affected by tracing measures, attentions should be paid to the coordination of isolation and tracing measures. For highly infectious public health incidents, even if the government adopts the most stringent isolation and tracing measures, the effects on repairing the negative impact of the epidemic on the economy is limited, and the attenuation of the policy is shown at the end of the curve.

Our model provides a theoretical framework for studying the effect of government policy intervention under public health emergencies, which has good scalability. Future researches can expand the policy toolbox on this basis and simulate the effects of fiscal policy, monetary policy, and macro-prudential policy. It can also be extended to a cross-border epidemic transmission model in an open economy to simulate economic risks, financial risks, capital flows and exchange rate fluctuations.

## Data Availability Statement

The original contributions presented in the study are included in the article/supplementary material, further inquiries can be directed to the corresponding author/s.

## Author Contributions

ZY: conceptualization, validation, writing original draft, and supervision. XC: visualization, project administration, and funding acquisition. ZW: software, resources, methodology, and data curation. LX: formal analysis, investigation, writing—review, and editing. All authors contributed to the article and approved the submitted version.

## Funding

We acknowledge the financial support from Shandong Provincial Natural Science Foundation (Grant Number: ZR2020QG032), Shandong Provincial Social Science Planning Office (Grant Numbers: 21DGLJ12; 21DJJJ02), Taishan Scholars Program of Shandong Province, China (Grant Numbers: ts201712059; tsqn201909135) and Youth Innovative Talent Technology Program of Shandong Province, China (Grant Number: 2019RWE004). All errors remain our own.

## Conflict of Interest

The authors declare that the research was conducted in the absence of any commercial or financial relationships that could be construed as a potential conflict of interest.

## Publisher's Note

All claims expressed in this article are solely those of the authors and do not necessarily represent those of their affiliated organizations, or those of the publisher, the editors and the reviewers. Any product that may be evaluated in this article, or claim that may be made by its manufacturer, is not guaranteed or endorsed by the publisher.
